# Dynamic plain abdominal film provides simple and effective diagnosis of delayed shunt insufficiency caused by abdominal adhesions after VP shunt

**DOI:** 10.1186/s41016-024-00378-z

**Published:** 2024-09-03

**Authors:** Zhiqiang Liu, Jintao Chen, Chaoqun Weng, Bei Liu, Zhixiong Lin

**Affiliations:** 1 Department of Neurosurgery, Fujian Sanbo Funeng Brain Hospital, Fuzhou, Fujian China; 2 Department of Neurosurgery, Sanbo Brain Hospital, Capital Medical University, Xiangshanyikesong 50#, HaiDian District, Beijing, China

**Keywords:** Hydrocephalus, VP shunt, Delayed, Shunt insufficiency

## Abstract

**Background:**

Many complications may occur after placement of the ventriculoperitoneal shunt (VP shunt) for hydrocephalus, and delayed shunt insufficiency (DSI) is among the most common. It is often caused by abdominal adhesions, which increases the difficulty of diagnosis. This study aimed to explore the clinical value of dynamic plain abdominal radiography (DPAR) as a simple diagnostic method for patients with DSI due to terminal adhesion of the peritoneal shunt after VP surgery.

**Methods:**

A total of 30 patients with high suspicion of DSI due to abdominal adhesions after VP surgery were included. DPAR was used for prospective assessment. The interval between the first and second PAR was 4–6 h before surgery. If two plain abdominal radiography at different times indicated that the end of the shunt tube in the abdominal segment was fixed, it was diagnosed as DSI due to adhesion of the shunt tube at the abdominal end. The peritoneal end of the shunt tube was surgically repositioned. Postoperative DPAR was repeated to evaluate the distance of the shunt outlet within the abdominal segment.

**Results:**

All cases showed clinical symptoms or imaging findings of shunt insufficiency. The diagnostic accuracy of DPAR was 96.67% (29/30). The end of the shunt tube in the abdominal segment of the preoperative group was fixed with abdominal plain film twice with a mean difference of 1.74 ± 1.18 cm. The mean postoperative change in the position of the end of the shunt tube in the abdominal section was 9.36 ± 2.64 cm, showing a significant difference compared with the preoperative group (*P* < 0.001). The mean postoperative EVANs index (0.37 ± 0.08) was significantly lower than the preoperative (0.42 ± 0.08) (*P* = 0.007), Glasgow coma scale score (12.8 ± 2.69) was higher than the mean preoperative score (11.36 ± 2.43) (*P* = 0.013).

**Conclusion:**

DPAR is a simple and effective method for the diagnosis of shunt insufficiency caused by delayed abdominal end adhesion after VP shunt.

## Background

The ventriculoperitoneal shunt (VP shunt) is still the most commonly used method for the effective treatment of hydrocephalus, but complications are as high as 20–40% in the first year after surgery [1] and may even reach 50% in children [2]. A least 60% of patients treated for hydrocephalus are expected to undergo a shunt modification during their lifetime [3], and the revision rate in children may even up to 80% [4]. Common complications include shunt infection, bleeding, shunt obstruction, ectopic shunt, excessive shunt, cerebrospinal fluid insufficiency, and shunt dependence, among others [5–7]. With the wide application of adjustable pressure shunt devices, excessive shunt is rare, and delayed shunt insufficiency (DSI) is quite common.

DSI is defined as the persistent hydrocephalus symptom caused by inadequate cerebrospinal fluid drainage [8]. The causes of DSI include shunt tube obstruction, improper pressure setting of the shunt valve, abdominal end factors, and other causes [5], among which abdominal end factors are the most difficult to identify. Abdominal end blockage is often caused by local adhesions [9]. To date, shunt tube radioisotope angiography [10], radionuclide angiography [11], abdominal CT, and other examinations have been performed, but all current methods are still difficult to determine accurately. Moreover, shunt angiography has the risk of causing infection, and the reliability of judging incomplete blockage is not high, so its clinical application has not been widely accepted. Abdominal CT is mainly used to determine whether there is local obvious encapsulated effusion, cyst formation, ectopic perforation, or other factors, but identification of peritoneal adhesion-induced shunt insufficiency is still not reliable, and multiple scanning radiation doses are high, increasing medical expenses. Therefore, effective inspection and diagnostic methods are still lacking. This study prospectively explored the clinical value of dynamic plain abdominal radiography (DPAR) as a simple diagnostic method for patients with DSI due to terminal adhesion of the peritoneal shunt after VP surgery.

## Methods

### Research hypothesis and strategy

**Fig. 1:**
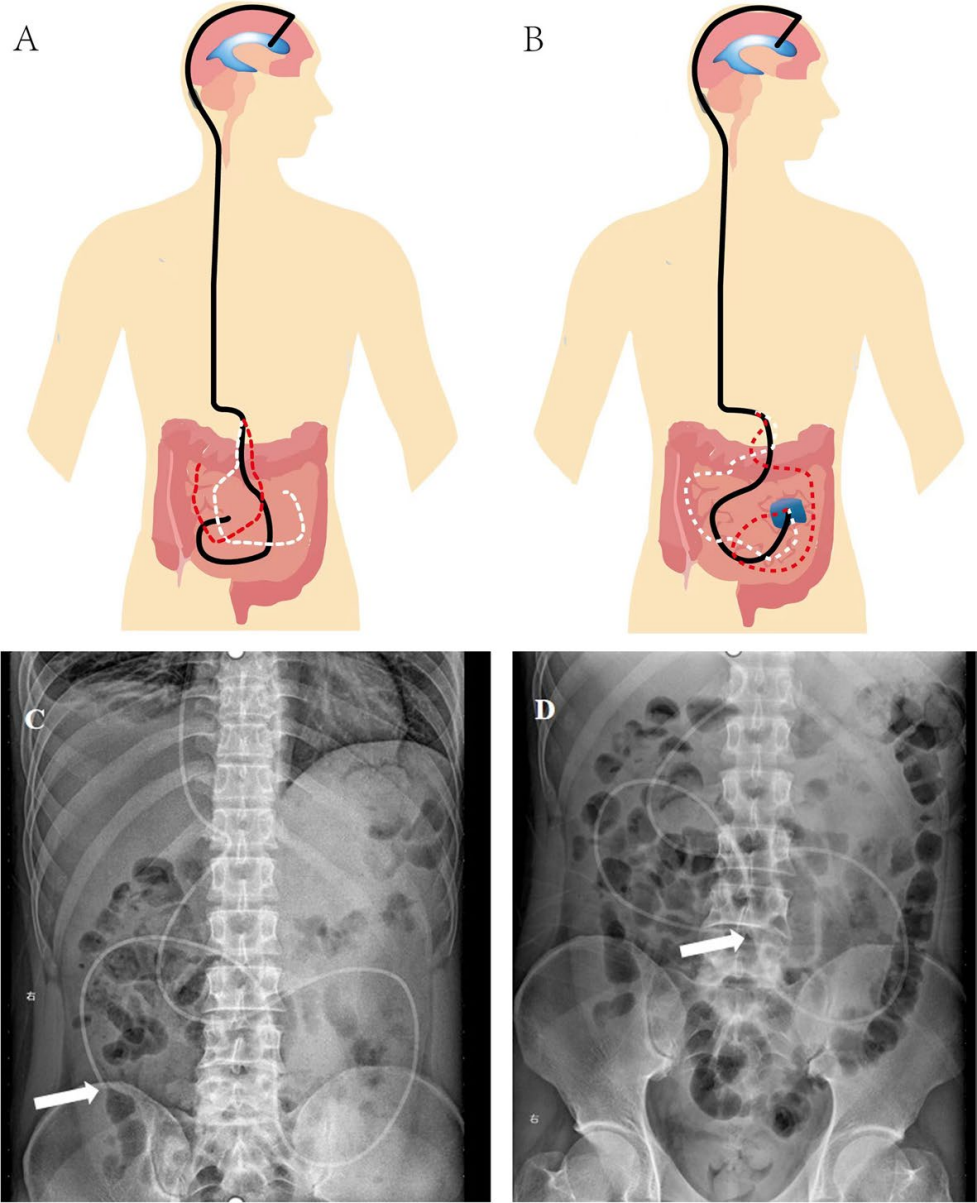
Diagram of the position of the end of the peritoneal end of the shunt tube over time and in patients with normal shunt function. **A** The position of the end of the peritoneal end of the shunt tube over time under normal conditions (different colors represent the position change). **B** The position of the end fixation does not change with time when there is local wrapping adhesion in the peritoneal end of the shunt tube. **C** A 34-year-old male patient underwent ventriculoperitoneal shunt due to right ventricular obstructive hydrocephalus. In 1 week post-operation, the shunt was functioning properly. The plain abdominal radiograph examination revealed the presence of the abdominal segment and the edge of the shunt. (arrow) **D **Upon reexamination of the plain abdominal radiograph 6 h later, a notable shift in the position of the end of the abdominal segment of the shunt tube was observed compared to its position 6 h prior (arrow)

According to the working principle of shunt tubes [12], intracranial pressure (ICP) = ventricle + peritoneal hydrostatic difference (HPD) + valve opening pressure (VOP) + peritoneal pressure (IAP) when using adjustable pressure tube shunt; and valve opening pressure (VOP) = intracranial pressure (ICP) difference between ventriculoperitoneal hydrostatic pressure (HPD) – anti-pressure device setting pressure (GD) – peritoneal pressure (IAP). The fluid that is normally shunted into the peritoneal cavity is easily absorbed. Therefore, the ventriculoperitoneal shunt model is considered to be an open system, and the cerebrospinal fluid entering the abdomen does not affect the intraperitoneal pressure. Consequently, the influence of hydrostatic pressure at the end is not considered in pressure regulation. However, when the end of the shunt duct in the abdominal segment is locally wrapped to form a local sac, the original open system becomes a limited sac system, and the original abdominal pressure is replaced by the pressure of the limited sac system, resulting in improving the hydrostatic pressure at the end. Therefore, the actual intracranial pressure is greater than the open cranial pressure, resulting in shunt insufficiency. In the early stage, the symptoms may be temporarily relieved by lowering the shunt valve pressure, but when the local cystic pressure at the end of the shunt peritoneal cavity is further increased, exceeding the minimum shunt pressure difference, the shunt no longer works properly. The local adhesion also makes the outlet of the shunt tube unable to change position. In general, the position of the end of the shunt tube in the abdominal segment changes with intestinal peristalsis (Fig. 1A, C, D), and motility at the end of the shunt tube is impaired due to local adhesion or wrapping (Fig. 1B). At the same time, most shunt tubes can be developed on X-ray. Based on this, we hypothesized that in patients with high suspicion of shunt insufficiency due to wrapping or abdominal adhesion, the diagnosis can be made by the DPAR showing a relatively constant local position of the shunt tube end in the abdominal segment.

### Study population

A total of 82 patients diagnosed with postoperative complications of VP performed for hydrocephalus who were admitted to our unit for surgical intervention from April 2017 to December 2023 were enrolled. All included patients provided signed informed consent to participate in the study.

**Fig. 2:**
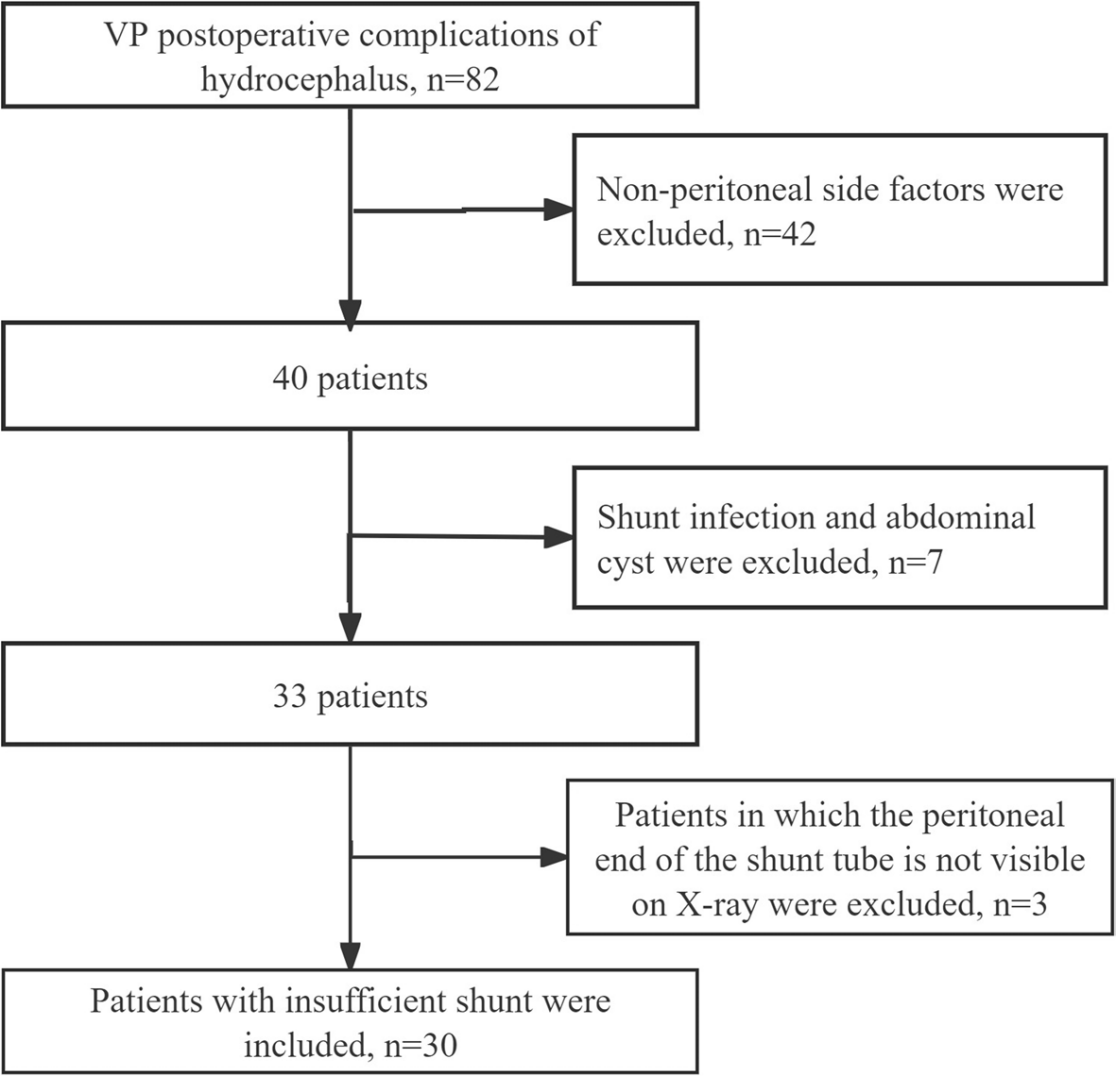
The flow chart of patient inclusion

The patient sample selection procedure is shown in Fig. 2. After screening according to inclusion and exclusion criteria, 30 cases with DSI were included, as shown in Fig. 2. Inclusion criteria were as follows: (1) patients with hydrocephalus who received VP shunt more than 1 week prior, with suspected shunt disorder; (2) clinical symptoms were obviously improved initially, and then became worse, such as headache or consciousness disorders and other neurological dysfunction; (3) CT or MRI showed ventricle enlargement or cerebral edema; (4) adjustable pressure shunt tube was adjusted to the lowest level, but without improvement; (5) the physical examination revealed that the shunt tube fluid storage bag exhibited good elasticity, the continuity of the shunt was not significantly compromised, and there was no evidence of local effusion in the subcutaneous tract of the shunt; (6) all men and non-pregnant women.

Exclusion criteria were as follows: (1) complicated with intracranial infection; (2) complicated with intracranial hemorrhage; (3) obstruction at the end of the ventricle; (4) shunt infection; (5) shunt valve failure; (6) shunt tube continuity disorder, such as rupture or tie or separation at the junction; (7) peritoneal cyst at the end of the shunt tube; (8) peritoneal infection; (9) shunt tube dependence syndrome; (10) excessive shunt; (11) shunt tube exposure; (12) shunt tube failed to develop on X-ray.

### Imaging technique

All patients were examined by DPAR with X-ray equipment (SHIMADZU Corp. Kyoto, Japan). The X-ray tube operated at 65 kV (peak) voltage, the tube current was 320 mA, and the X-ray volume was 51.2 mAs.

### Imaging evaluation

Imaging assessments were conducted independently by two radiologists each with 8 years of experience on standard PACS workstations. In order to facilitate proper abdominal peristalsis for the propulsion of potential shunt ends, DPAR was performed again 4-6 h (A working time zone) after the first radiography.

**Fig. 3:**
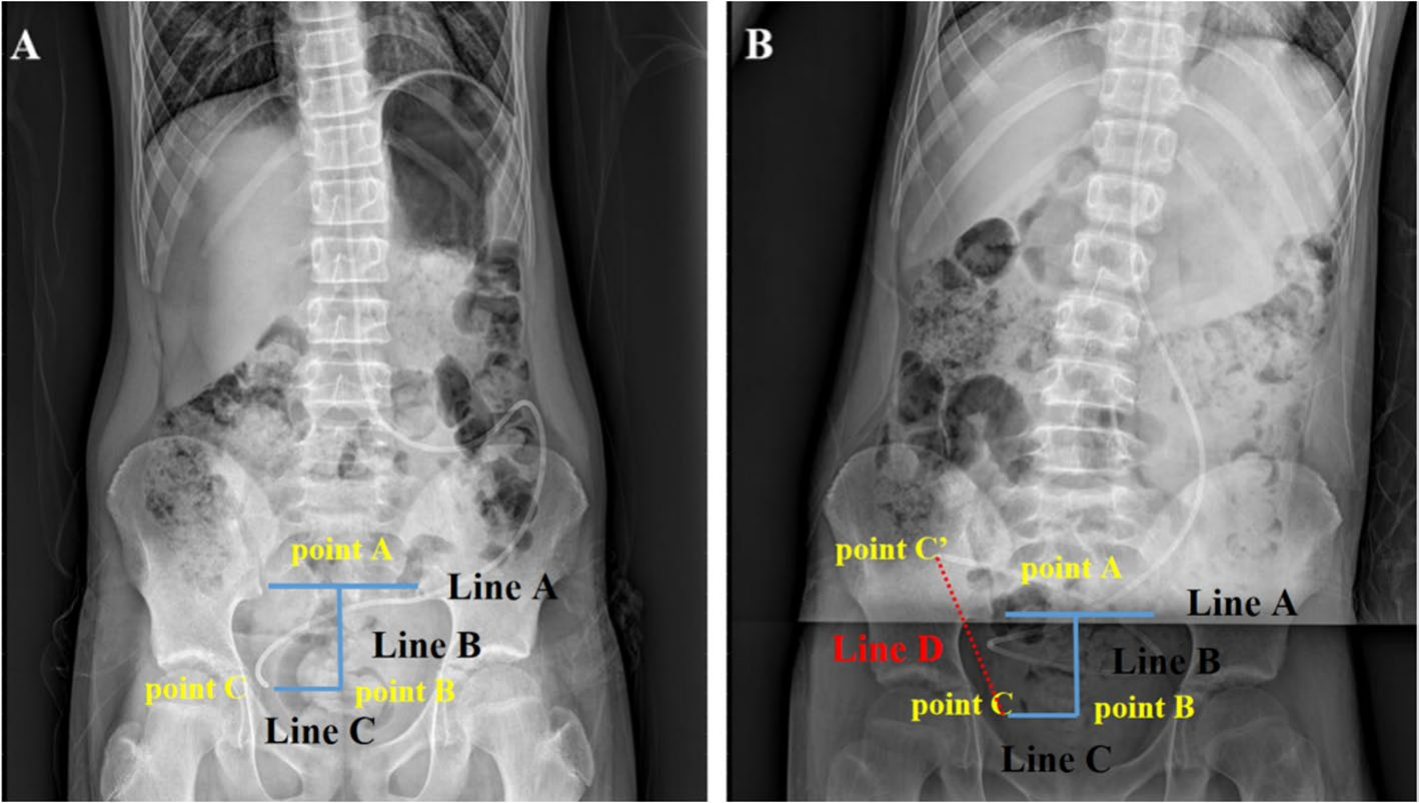
Comparison of peritoneal shunt end position changes and numerical methods to measure the distance of position changes. **A** The midpoint (point A) of the line connecting the lower margin of the sacroiliac joint (line A) on the first plain abdominal film was measured. At point A, A vertical line (line B) was made with line A, and then the vertical line (line C) between the end of the shunt tube in the abdominal segment (point C) and line B was made. The distance between line C and line B was separately measured. **B** In the same method, point C was marked on the second plain abdominal film 6 h later, and the position of the end of the shunt tube in the upper abdominal segment of the second plain abdominal film was marked as point C’, and the straight-line distance between point C and C’ was calculated from line D

Changes in position at the end of the shunt tube in the abdominal segment were compared, and the distance values of the position changes were measured, as shown in Fig. 3. The initial step in the measurement process involved identifying and marking the midpoint (point A) of the inferior border of the sacroiliac joint junction line (line A) on the first plain abdominal radiograph. Subsequently, a vertical line (line B) was extended from point A, followed by the drawing of another vertical line (line C) connecting the end of the abdominal segment of the shunt (point C) to line B. The point of intersection between line B and line C was subsequently identified and marked as point B. The distance between point B and point C was then measured, as illustrated in Fig. 3A. Based on the parameters and methods assessed in the initial plain abdominal radiograph, point C was identified on the subsequent plain abdominal radiograph 6 h later. Subsequently, the location of the end of the abdominal segment shunt on the subsequent plain abdominal radiograph was designated as point C’, and the linear distance between points C and C’—referred to as line D—was determined and recorded (see Fig. 3B). The PACS workstation was utilized for objective measurement utilizing its proprietary software. To mitigate the impact of varying angles in the visual angle collection on judgment results, three fixed reference points and three standard lines were established during the measurement process. This allowed for the evaluation of the distance between the end of the shunt tube and the three lines. The resulting values were averaged based on independent measurements conducted by two assessors.

### Treatment method

Abdominal exploration was performed, and the shunt distal tubing was adjusted. After dredging the abdominal end of the shunt catheter, the cerebrospinal fluid drainage was examined to ensure it was unobstructed; another small incision was made in the abdomen to re-insert the abdominal end of the shunt catheter into the abdominal cavity. Since the adhesion area at the end of the shunt is a limited space formed by the greater omentum wrapping, and this wrapping can be around the channel formed by the shunt to the abdominal wall incision, a new incision must be made 3CM away from the original incision when adjusting the shunt to avoid entering the limited space along this channel when re-catheterizing. Abdominal shunt adjustments performed in accordance with standard postoperative care do not require specialized care.

### Reference standard

A neurosurgeon with 10 years of experience evaluated each patient’s clinical data, including hydrocephalus etiology, symptoms, clinical examination, intraoperative findings, CT, and X-ray images.

Verification criteria were as follows: an exploratory operation was performed at the peritoneal end of the shunt tube to determine whether the exit of the peritoneal end was obstructed, resulting in poor cerebrospinal fluid drainage of the shunt tube.

Efficacy judgments were as follows: (1) effective, improvement of clinical symptoms—headache, vomiting, disturbance of consciousness, and other clinical symptoms improved or Glasgow Coma Scale (GCS) score increased. Imaging improvement, cranial CT showed relief of ventricular pathologic dilatation or cerebral edema. If the first two items are not satisfied, changes in the position of the shunt outlet in the abdominal section are shown by the dynamic abdominal plain film. (2) Ineffective, no improvement in clinical symptoms or CT imaging.

### Statistical analysis

Categorical variables, including gender, clinical symptoms, etiological frequency, and percentage, are presented as numbers and percentages. Quantitative data are expressed as the mean plus standard error (SEM) of the mean. Graphic images were generated on a bioinformatics platform. Statistical analysis for multiple group data was performed using the Analysis of Variance (ANOVA) method followed by the Students *t*-test or Wilcoxon rank sum test to compare data between the two groups. Differences in EVAN scores and GCS scores before and after surgery, as well as differences in the position of the end of the shunt tube in the abdominal cavity between the under-shunt group before surgery and the negative patency group after catheterization, were analyzed. Statistical analyses were performed using the GraphPad Prism (version 9.1.0, San Diego, CA), and *P* < 0.05 was considered statistical significance.

## Results

### Patients’ baseline data

**Fig. 4:**
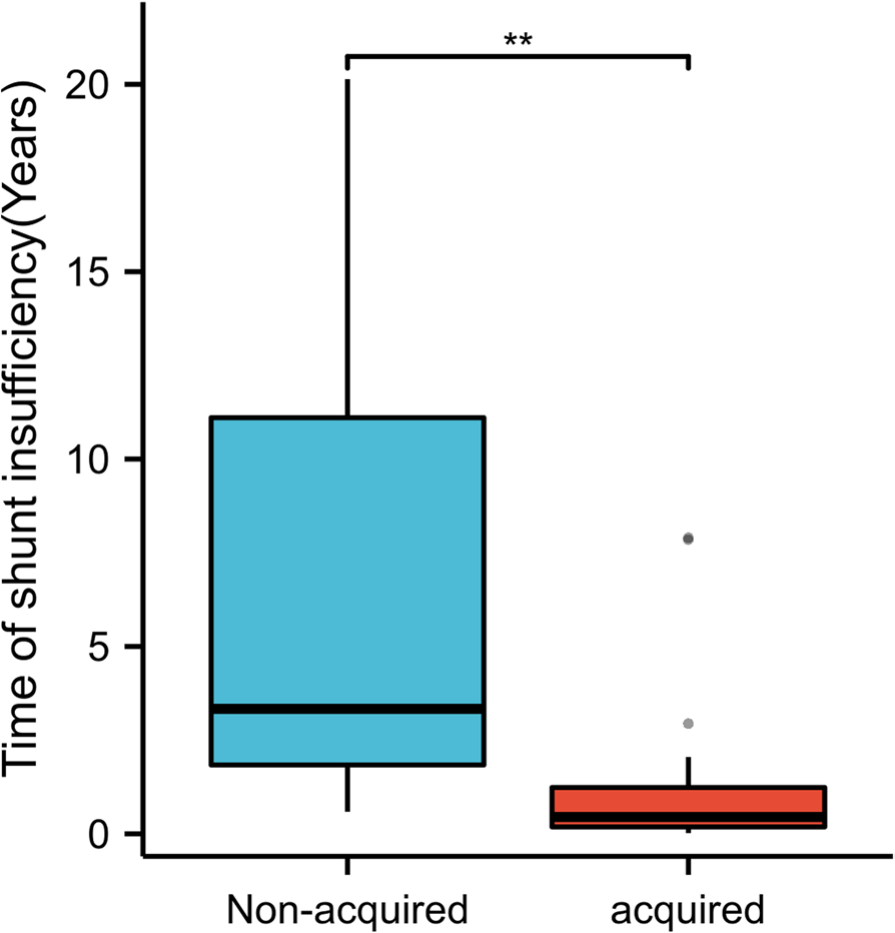
Time and diagnosis of delayed shunt insufficiency after first VP shunt. Time of shunt insufficiency after the first VP shunt surgery. The time of insufficient shunt in patients with congenital hydrocephalus was significantly later than that of tumor, trauma, hemorrhage, and other types of hydrocephalus (Wilcoxon rank sum test, *P* = 0.002) (**P < 0.01)

The study included 30 patients, including 16 males (53.3%) with a mean age of 42.1 ± 24.7 years, a range of 9–82 years; and 14 females (46.7%) with a mean age of 56.9 ± 16.3 years, a range of 18–79 years. Shunt insufficiency varied between patients, occurring from 2 weeks to 20 years after VP shunt surgery (mean 2.95 ± 5.09 years). In 20 cases, shunt inefficiency occurred within 2 years after the VP shunt. Based on the classification of hydrocephalus etiology, the times of shunt insufficiency after the first VP shunt are shown in Fig. 4. DSI occurrence times of unacquired hydrocephalus after the first VP shunt were longer than those of acquired hydrocephalus (*P* < 0.01), suggesting that the problem of under-shunt of secondary hydrocephalus in the short term requires extra attention. Patients’ clinical characteristics are shown in Table 1.

**Table 1:** Clinical data of hydrocephalus patients

**Factors**		**Numbers/proportions (%)**
Gender		
	Male	16 (53.3%)
	Female	14 (46.7%)
Age (years)		49 ± 22.14
Time of shunt insufficiency after VP shunt (years)		2.95 ± 5.09
	Within 2 years after VP operation (cases)	20 (66.67%)
Clinical symptoms		
	Disturbance of consciousness	18 (60%)
	Symptoms of cranial hypertension	11 (36.67%)
	Speech dysfunction	2 (6.67%)
	Binocular blurred vision	1 (3.33%)
	Augmentation outside the bone window	1 (3.33%)
	Evans Index (pre-operation)	0.42 ± 0.08
	Evans Index (post-operation)	0.37 ± 0.08
	GCS score before adjustment of shunt	11.36 ± 2.43
	GCS score after adjustment of shunt	12.8 ± 2.69
Etiology of hydrocephalus		
	Tumor	6 (20%)
	Trauma	7 (23.33%)
	Hemorrhage	8 (26.67%)
	Infection	1 (3.33%)
	Non-acquired hydrocephalus	8 (26.67%)
Position change of peritoneal segment of shunt tube		
	Preoperative group	1.74 ± 1.18 cm
	Postoperative and negative groups	9.36 ± 2.64 cm

### Diagnostic accuracy of dynamic plain abdominal radiography

**Fig. 5:**
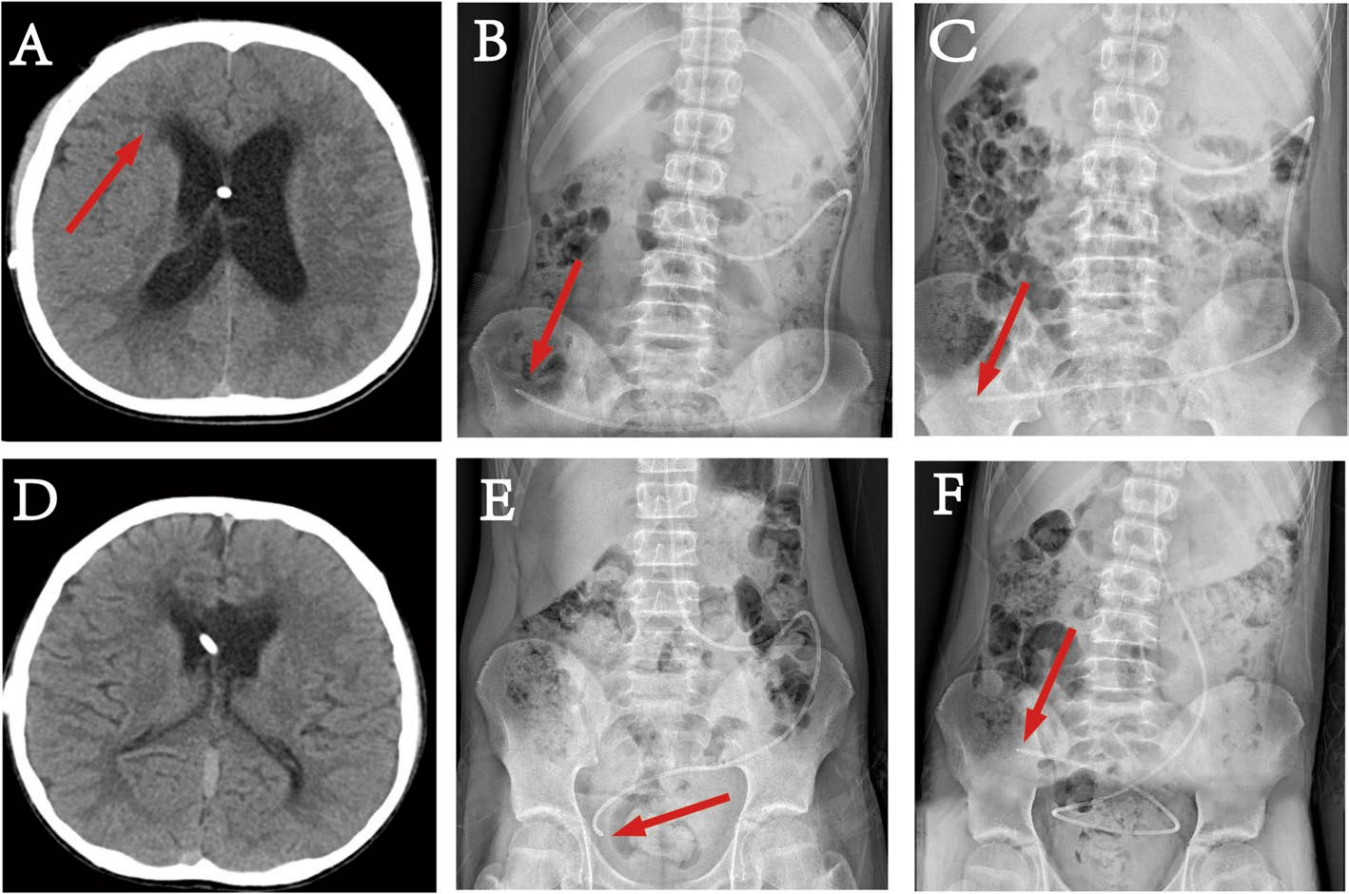
Case presentation before and after shunt adjustment of the abdominal segment of the shunt in patients with shunt insufficiency due to abdominal segment factors after ventriculoperitoneal shunt. **A**–**F** Male, 11 years old: Right lateral ventriculoperitoneal shunt was performed due to “three-ventriculoposterior space-occupying lesions with hydrocephalus.” Eight years after the first surgery, he complained of “intermittent headache for 1 month, aggravated with vomiting for 5 days.” **A** CT before the adjustment of the abdominal end of the shunt catheter after VP showed bilateral dilatation of the lateral ventricle, interstitial edema of the frontal angle of the lateral ventricle (red arrow), and swelling of brain tissue. **B** The first anteroposeural X-ray of the abdomen before adjustment of the shunt abdominal end shows the position of the end of the shunt abdominal end (red arrow). **C** The second anteroposeural X-ray of the abdomen before the adjustment of the abdominal end of the shunt (reviewed 5 h after the first X-ray) showed that the position of the distal end of the abdominal end of the shunt was fixed and unchanged (red arrow). **D** CT after the adjustment of the abdominal end of shunt catheter showed that bilateral lateral ventricles were smaller than before, interstitial brain edema was reduced, and brain tissue swelling was reduced. **E** The position of the end of the abdominal end of the shunt catheter (red arrow) was shown in the first anteropoetal abdominal X-ray after the adjustment of the abdominal end of the shunt catheter. **F** The second anteropoetal X-ray of the abdomen after the adjustment of the abdominal end of the shunt shows the change in the position of the abdominal end of the shunt (red arrow)

DPAR of 30 patients showed that the abdominal end of the shunt catheter was fixed (Fig. 5B, E), and the shunt end adhesions caused by the shunt catheter were diagnosed as shunt insufficiency.

**Fig. 6:**
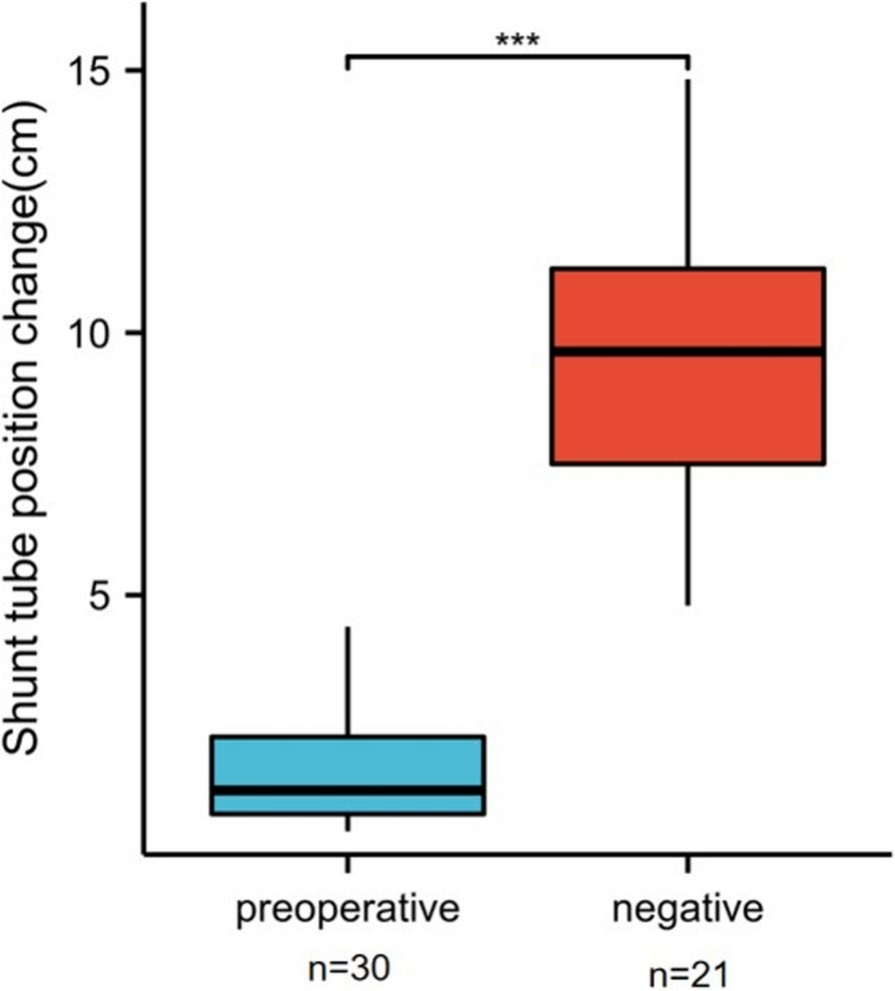
Comparison of the changes in the position of the end of the abdominal segment shunt between patients with shunt insufficiency and patients with normal shunt function after ventriculoperitoneal shunt for hydrocephalus. The change distance of the end of shunt peritoneal cavity in the negative group was significantly greater than that in the preoperative group (Wilcoxon rank sum test, *P* < 0.001) (***P < 0.001)

All patients underwent a second VP shunt operation. In 28 cases, the abdominal end catheters were pulled out and a small amount of retinal fats or cellulose membrane-like tissues were wrapped at the end. Fibrin-like tissue blocked the end of the shunt catheter in one case. During the operation, local adhesions or blocked tissues were removed, and cerebrospinal fluid (CSF) was automatically drained out, or CSF was automatically drained out after the fluid storage sac was pressed again. The automatic drainage of CSF was observed to be smooth, and the shunt catheter was re-inserted. In the other one case, no tissue blockage and no improvement in postoperative symptoms and imaging were observed, for which the cause was finally confirmed to be poor ventricular drainage, and which was cured after VP with a shunt catheter inserted in the other ventricle. After the second VP shunt operations, CT imaging showed the patients’ ventricles to have become smaller, indicating adequate drainage (Fig. 5D). The DPAR showed changes in the position of the shunt catheter at the abdominal end (Figs. 5C, F). The diagnostic accuracy of the DPAR was 96.67% (29/30). Comparisons were made between the positive group of preoperative shunt tube terminal position changes and the negative group of postoperative shunt tube patency. The end of the shunt tube in the abdominal segment of the preoperative group was fixed with DPAR at two different times, and the change was 1.74 ± 1.18 cm. Compared with the postoperative and negative groups, the change in the position of the end of the shunt tube in the abdominal section at different times was 9.36 ± 2.64 cm, which showed a significant difference compared with changes in the preoperative group (*P* < 0.001) (Fig. 6).

### Shunt surgery outcomes

**Fig. 7:**
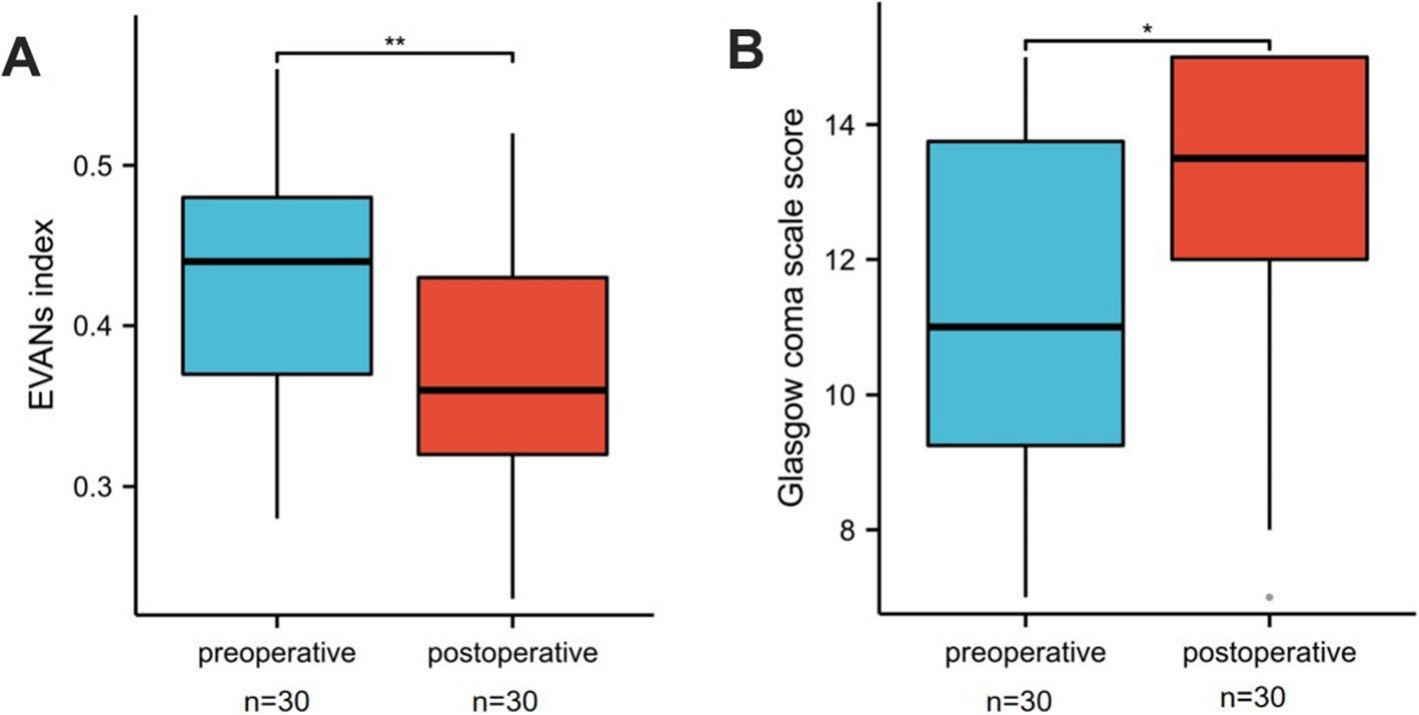
Outcomes after the second operation for shunt insufficiency. **A** Postoperative Evans index was lower than the preoperative Evans index (independent-samples *t* test, *P* = 0.007)(**P < 0.01). **B** Postoperative Glasgow coma scale score was higher than preoperative Glasgow coma scale score (Wilcoxon rank sum test, *P* = 0.013) (*P < 0.05)

Patients’ follow-ups ranged from 3 months to 2 years. The clinical symptoms of 20 patients were improved, while the clinical symptoms of 3 patients were not improved, but the imaging was improved. The postoperative Evans index (0.37 ± 0.08) was significantly lower than the preoperative Evans index (0.42 ± 0.08) (*P* = 0.007), and the GCS score (12.8 ± 2.69) was significantly higher than preoperative scores (11.36 ± 2.43) (*P* = 0.013) (Fig. 7).

DPAR is a simple and effective method to identify shunt insufficiency caused by delayed abdominal adhesion in patients with hydrocephalus after VP surgery.

## Discussion

The gastrointestinal tract is the only internal organ with its own independent nervous system—the enteric nervous system (ENS) [13, 14]. Under the control of the ENS, the functions of the small intestine include segmental movement and peristalsis, mixing, stirring, and pushing the intestinal contents to the distal end, forming migrating motor complexes (MMC) [15, 16]. The small intestine is constantly moving autonomously, and when the shunt catheter is not adhered to or wrapped by external actions, fixing its position should be difficult and it should remain constantly changing. In the present study, we observed changes in the position of the shunt when abdominal radiographs were taken in patients with functioning shunt catheters (Figs. 5E, F). When the end of the shunt catheter was wrapped by external adhesions, the peritoneal terminal movement with intestinal peristalsis was inevitably limited (Figs. 5B, C). Therefore, based on this principle, we performed DPAR in patients who were clinically suspected to have local adhesions at the shunt abdominal end and had clinical symptoms caused by shunt insufficiency. Therefore, when the position of the catheter is fixed, plain film supports the adhesion of the shunt abdominal end accompanied by clinical manifestations of aggressive hydrocephalus, and an exploration of the shunt abdominal end should be performed.

The key point of the operation is to open the original abdominal incision, remove the peritoneal segment of the shunt tube, and remove the adherent tissue at the end of the peritoneal end of the shunt tube, which consists mostly of omental adipose tissue or fibrous tissue. Then a new surgical incision is made in the abdomen, and the peritoneal end of the shunt tube is re-inserted into the abdominal cavity to avoid re-entering the original wrapped area from the original pseudo-channel. In the present group of patients (29 cases), clinical symptoms improved after surgery, which demonstrated the correctness of the preoperative judgment. It should be noted that a small number of patients with intraperitoneal adhesion of the shunt have an insufficient shunt and incomplete blockage. The brain CT in one case in the present study showed no significant increase in hydrocephalus, but the patient’s clinical symptoms were aggravated, leading us to consider the possibility of shunt deficiencies. In particular, the clinical symptoms improved in patients who underwent daily compression of the fluid storage sac to increase drainage. Combined with the DPAR showing abdominal end fixation, it became more important to perform abdominal end exploration of the shunt. The shunt adjustment procedure involves a minimally invasive incision of approximately 2–3 cm, with minimal trauma and a brief operation duration, making it suitable for use in different hospitals. While some scholars may advocate for laparoscopic examination of the peritoneal segment shunt, our perspective is that the majority of neurosurgeons lack proficiency in laparoscopic surgery techniques and that its utilization would result in increased surgical costs. This issue can be addressed by simply clearing the end of the abdominal segment of the shunt tube and re-catheterizing to prevent the original false channel from entering the adhesion area and compromising the functionality of the shunt.

From the VP of distal adhesion that occurred after the first VP shunt surgery (Fig. 4), the secondary hydrocephalus may appear earlier than abdominal cavity adhesions and may also be related to factors such as hemorrhage, tumor, drainage of cerebrospinal fluid after changes. The secondary hydrocephalus may be more likely to induce local retinal tissue on the abdominal cavity shunt catheter package and should be associated with the physiological functions of the greater omentum, as the inflammatory reaction of a foreign body [17].

Based on current knowledge, the common causes of obstruction at the abdominal end of shunt are as follows: (1) local adhesions, cellulose tissue and/or greater omentum tissue wrap; (2) aseptic celiac cyst formed locally in the abdominal cavity [18]. Due to the space-occupying effect, patients often present with abdominal pain and distension, which may be accompanied by fever, abdominal tenderness, and acute abdominal symptoms in some patients [3]. Abdominal CT or ultrasound can assist in diagnosis; (3) the shunt pipe is crimped or broken. Abdominal X-ray examination may also be helpful in the diagnosis of this cause; (4) abdominal infection.

In addition to the present cases, it is suggested that when clinical symptoms of shunt insufficiency appear and infection is excluded, performing the following tests will help identify the cause of shunt insufficiency: (1) by pressing the shunt catheter valve liquid storage sac to understand the degree of elasticity, preliminary screening to determine whether there is the shunt catheter ventricle end, and the valve or the distal end of the shunt catheter blockage [19, 20]. (2) By checking whether any abnormality is found in the path of the body surface of the shunt catheter, the obvious disconnection or fracture of the catheter can be excluded. (3) If the adjustable pressure shunt catheter is used, retesting the valve pressure of the shunt catheter will help to eliminate the abnormal shift [21, 22]. (4) Performing a CT scan excludes intracranial hemorrhage, over-drainage, shunt catheter ventricular end ectopia, and other abnormalities [22–24]. (5) To understand the intracranial pressure through lumbar puncture, and check the routine and biochemical examination of cerebrospinal fluid to exclude abnormal cerebrospinal fluid. (6) Investigate the abdominal end adhesion and fixation of the shunt by dynamic abdominal anteropotentional X-ray. (7) Use X-ray inspection to rule out shunt catheter fracture, discount, valve, and shunt catheter separation [25]. (8) When there is no apparent rupture of the catheter, it is necessary to further add an ultrasound examination to exclude possible events that are not detectable with plain radiography and that can interfere with the function of the shunt, such as cysts at the tip, malposition, or others [26, 27].

## Limitations

This research is a single-center study with a limited number of cases, which may affect the generalizability of our findings. The small sample size also impacts the statistical power and robustness of the results. Additionally, potential selection bias may affect the study’s validity. Future research will include multi-center studies with larger and more diverse populations to address these limitations and validate our findings across different clinical settings.

## Conclusion

DPAR provides better diagnostic significance in evaluating shunt insufficiency caused by local adhesions and blockage at the abdominal end of shunt catheter, for which it is worth promoting regular clinical application.

## Data Availability

The datasets used and/or analyzed during the current study are available from the corresponding author on reasonable request.

## Abbreviations

CSF: Cerebrospinal fluid
VP: Ventriculoperitoneal
GCS: Glasgow coma scale
