# Correction: Dynamic plain abdominal film provides simple and effective diagnosis of delayed shunt insufficiency caused by abdominal adhesions after VP shunt

**DOI:** 10.1186/s41016-024-00382-3

**Published:** 2024-10-16

**Authors:** Zhiqiang Liu, Jintao Chen, Chaoqun Weng, Bei Liu, Zhixiong Lin

**Affiliations:** 1 Department of Neurosurgery, Fujian Sanbo Funeng Brain Hospital, Fuzhou, Fujian China; 2 Department of Neurosurgery, Sanbo Brain Hospital, Capital Medical University, Xiangshanyikesong 50#, HaiDian District, Beijing, China

**Correction** **:** **Chinese Neurosurgical Journal 10, 26 (2024)**

**https://doi.org/10.1186/s41016-024-00378-z**

Following publication of the original article [1], the authors reported that the order of the references needed to be modified.

The original article [1] has been corrected.
